# Trypanin, a Component of the Flagellar Dynein Regulatory Complex, Is Essential in Bloodstream Form African Trypanosomes

**DOI:** 10.1371/journal.ppat.0020101

**Published:** 2006-09-29

**Authors:** Katherine S Ralston, Kent L Hill

**Affiliations:** 1 Department of Microbiology, Immunology, and Molecular Genetics, University of California Los Angeles, Los Angeles, California, United States of America; 2 Molecular Biology Institute, University of California Los Angeles, Los Angeles, California, United States of America; Harvard School of Public Health, United States of America

## Abstract

The Trypanosoma brucei flagellum is a multifunctional organelle with critical roles in motility, cellular morphogenesis, and cell division. Although motility is thought to be important throughout the trypanosome lifecycle, most studies of flagellum structure and function have been restricted to the procyclic lifecycle stage, and our knowledge of the bloodstream form flagellum is limited. We have previously shown that trypanin functions as part of a flagellar dynein regulatory system that transmits regulatory signals from the central pair apparatus and radial spokes to axonemal dyneins. Here we investigate the requirement for this dynein regulatory system in bloodstream form trypanosomes. We demonstrate that trypanin is localized to the flagellum of bloodstream form trypanosomes, in a pattern identical to that seen in procyclic cells. Surprisingly, trypanin RNA interference is lethal in the bloodstream form. These knockdown mutants fail to initiate cytokinesis, but undergo multiple rounds of organelle replication, accumulating multiple flagella, nuclei, kinetoplasts, mitochondria, and flagellum attachment zone structures. These findings suggest that normal flagellar beat is essential in bloodstream form trypanosomes and underscore the emerging concept that there is a dichotomy between trypanosome lifecycle stages with respect to factors that contribute to cell division and cell morphogenesis. This is the first time that a defined dynein regulatory complex has been shown to be essential in any organism and implicates the dynein regulatory complex and other enzymatic regulators of flagellar motility as candidate drug targets for the treatment of African sleeping sickness.

## Synopsis

African trypanosomes are protozoan parasites that cause African sleeping sickness, a fatal disease with devastating health and economic consequences. These parasites are indigenous to a 9 million-km^2^ area of sub-Saharan Africa where 60 million people live at risk of infection every day. In addition to the tremendous human health burden posed by trypanosomes, their infection of wild and domestic animals presents a barrier to sustained economic development of vast regions of otherwise productive land. Current drugs used for treatment of sleeping sickness are antiquated, toxic, and often ineffective; thus, there is a dire need for the development of innovative approaches for therapeutic intervention. Trypanosomes are highly motile and this motility requires coordinated regulation of axonemal dynein, a molecular motor that drives beating of the parasite's flagellum. In the present work, the authors demonstrate that the protein trypanin, which is part of a signaling system that regulates the flagellar dynein motor, is essential in bloodstream stage African trypanosomes. This surprising finding raises the possibility that numerous enzymes and regulatory proteins that are necessary for flagellar motility may represent novel targets for chemotherapeutic intervention in African sleeping sickness.

## Introduction

African trypanosomes, e.g. Trypanosoma brucei and related subspecies, are protozoan parasites that cause African trypanosomiasis in humans and animals. T. brucei is the causative agent of human African trypanosomiasis, a fatal disease that is commonly referred to as “African sleeping sickness.” In the last three decades there has been a dramatic rise in the incidence of human sleeping sickness and it is estimated that 500,000 new infections occur annually [1,2]. Current drugs used for treatment of sleeping sickness are antiquated, toxic, and often ineffective; thus, there is a dire need for the development of innovative approaches for therapeutic intervention. T. brucei is transmitted via a tsetse fly vector and alternates between bloodstream form and insect form (procyclic) lifecycle stages, which are adapted to survive in mammalian hosts and tsetse flies, respectively. These parasites are highly motile in both lifecycle stages and motility is suspected to be important for parasite development in the tsetse fly, as well as pathogenesis in the mammalian host [3].

Motility in trypanosomes is mediated by a single flagellum that emerges from the flagellar pocket at the posterior end of the cell. The flagellum consists of a canonical 9 + 2 axoneme, together with a filamentous paraflagellar rod (PFR), which is attached to and runs alongside the axoneme [4]. The flagellum is surrounded by its own membrane and is attached to the cell body along most of its length by a series of regularly spaced, electron-dense fibers that connect the axoneme and PFR to a cytoplasmic filament subtending the plasma membrane [3,5]. Together with a quartet of specialized subpellicular microtubules, these structures comprise a flagellum attachment zone (FAZ) that extends from the flagellar pocket to the anterior end of the cell [6]. Transmission electron microscopy studies indicate, to a first approximation, that the T. brucei axoneme contains many of the ultrastructural features observed in other eukaryotic flagella, such as radial spokes, central pair projections, dynein arms, and nexin links [3,7,8]. Recent work using RNA interference (RNAi) in procyclic cells has demonstrated functional conservation for a few axonemal proteins and confirmed that both the axoneme and PFR are critical for normal cell motility [9–12].

In addition to its function in cell motility, the flagellum is critical for host-parasite interaction [13], cellular morphogenesis [14,15], organelle positioning [16], and cell division [5,12,14,16,17]. Of particular interest is recent work demonstrating that the flagellum and FAZ provide structural and positional cues that influence cytokinesis and cell morphogenesis [14,15,18]. Disrupting flagellum attachment results in a failure to initiate cytokinesis and is lethal [17,19,20]. Blocking flagellum biogenesis by RNAi knockdown of intraflagellar transport components results in cells in which the new flagellum and FAZ are shorter than normal [14]. These cells appear to initiate cytokinesis at a point that correlates with the position of the truncated FAZ filament, giving rise to progressively shorter cells in the culture and ultimately yielding aflagellate cells that are nonviable.

Although flagellum formation and attachment are critical, simply having an attached flagellum is not sufficient to allow normal cell division in procyclic cells. RNAi knockdown of radial spoke and central pair proteins leads to cells with full-length, attached flagella that exhibit only a rudimentary beat [12]. Flagellar “paralysis” in these mutants is invariably followed by a failure in the final stages of cytokinesis, with cells accumulating as multicellular clusters attached at their extreme posterior ends. Wild-type cells actively rotate relative to one another at this stage and it appears that physical forces provided by flagellar beating are required for final cell separation, since mechanical agitation of paralyzed mutants rescues the cytokinesis defect [12].

The *T. brucei* flagellum is a multifunctional organelle and exhibits several unusual features that might be exploited as drug targets [3,5,7]. However, most of the work on flagellum structure-function in T. brucei has been conducted on the procyclic lifecycle stage and much less is known in the bloodstream stage. Moreover, almost all of the work conducted to date has focused on structural components of the axoneme and PFR, with very little known about enzymatic and regulatory activities that drive flagellar beat. One regulatory component is trypanin, a 54-kDa coiled-coil protein that is highly conserved among organisms with motile flagella [21]. In procyclic cells, trypanin is localized along the length of the flagellum and functions as part of a flagellar dynein regulatory complex (DRC) that regulates axonemal dynein in response to signals from the central pair apparatus [10,12,21,22]. The human trypanin homologue binds microtubules directly and thus may link the DRC to the axoneme (K. Hill and R. Crosbie, unpublished data). Trypanin RNAi knockdown in procyclic T. brucei results in misregulation of flagellar beat and loss of directional cell motility, although these mutants remain viable [10]. Here we investigate the requirement of this flagellar dynein regulatory system in bloodstream form trypanosomes. We demonstrate that trypanin is localized to the flagellum of bloodstream form trypanosomes with a pattern that is indistinguishable from that seen in procyclic cells. Surprisingly, RNAi knockdown of trypanin in bloodstream cells is lethal. Trypanin-deficient mutants fail to initiate cytokinesis, but continue through multiple rounds of mitosis and kinetoplast replication, ultimately accumulating as large, amorphous cellular masses with multiple nuclei, kinetoplasts, flagella, and FAZ structures. These results implicate the DRC and other regulators of flagellar beat as candidate drug targets in trypanosomes and emphasize the dichotomy between factors that contribute to cell division and cell morphogenesis in different lifecycle stages of these pathogens.

## Results

### Trypanin Is Essential in Bloodstream Form African Trypanosomes

**Figure 1:**
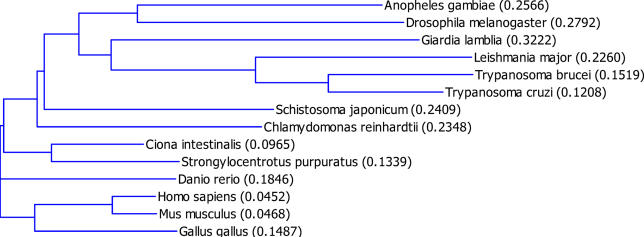
TPN Is Broadly Conserved Among Organisms with Motile Flagella Dendrogram of TPN-related proteins. The degree of divergence [41] between sequences is given in parentheses. TPN homologues were not found in organisms that lack flagella/cilia, or that only possess immotile cilia.

We previously reported that trypanin represents a broadly conserved protein family that is represented in several divergent eukaryotes [21]. We conducted a more thorough phylogenetic analysis of trypanin homologues using currently available genome databases (Figure 1). A common feature of all organisms having trypanin homologues is that these organisms are capable of synthesizing motile flagella. Trypanin homologues were not identified in the genomes of organisms that lack flagella, e.g. fungi, slime molds and vascular plants. We also did not find any trypanin-homologous sequences in the genomes of Caenorhabditis elegans or *C. briggsae,* which only have immotile sensory cilia. This phylogenetic distribution is consistent with trypanin's function as part of an axonemal DRC [12,22]. The finding that trypanin is present in organisms as diverse as Giardia lamblia and humans suggests that the DRC originated very early in eukaryotic evolution.

**Figure 2:**
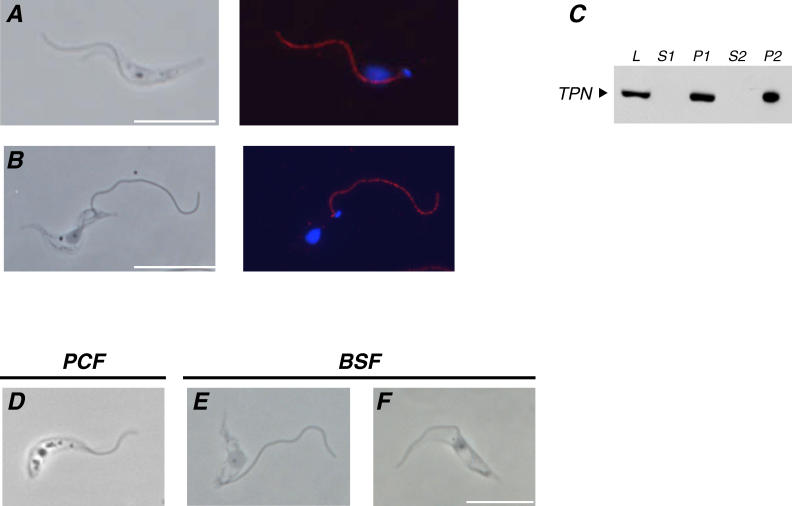
TPN Localizes to the Flagellum in Procyclic and Bloodstream Form Trypanosomes (A–C) Immunofluorescence of procyclic (A) and bloodstream form (B) cells. Cytoskeletons were prepared by detergent extraction and used for immunofluorescence with anti-TPN monoclonal antibodies. Phase-contrast images are shown in the left panels and merged images are shown in the right panels with antibody staining in red and DAPI staining in blue. (C) Western blot showing that TPN is quantitatively associated with the flagellar cytoskeleton (P2). Whole cell lysates (L), or the indicated subcelluar fractions were prepared as described [21] from bloodstream form cells. Intact cytoskeletons (P1) were separated from detergent-soluble proteins (S1) by centrifugation at 1,000 × g, then further extracted with 0.5 M NaCl to depolymerize the subpellicular cytoskeleton, leaving the flagellar cytoskeleton (P2) intact. The intact flagellar cytoskeleton (P2) was separated from solubilized proteins (S2) by centrifugation at 16,000 × g. (D–F) Cytoskeletons from procyclic (D) and bloodstream form (E and F) cells, prepared by extraction with 1% NP-40 to remove cellular membranes as described [21,45]. (D and E) Cells were extracted in solution then spotted onto slides and viewed directly. (F) Cells were adhered to glass coverslips prior to detergent-extraction. Scalebars are 10 μm.

In procyclic cells, trypanin is localized along the length of the flagellum and functions as part of a dynein regulatory system that regulates flagellar beat in response to signals from the radial spokes and central pair apparatus [10,12,21,22]. Immunofluorescence of bloodstream form cells demonstrated that trypanin exhibits a punctate distribution along the length of the flagellum that is indistinguishable from that seen in procyclic cells (Figure 2A and 2B). Likewise, biochemical fractionation demonstrates that trypanin fractionates quantitatively with the flagellar cytoskeleton (Figure 2C), as shown previously for procyclic-form cells [21]. The flagellum of bloodstream form cells prepared for immunofluorescence was always detached from the cell body. Indeed, simply removing cellular membranes with non-ionic detergent caused flagellum detachment in bloodstream form cells, while the flagellum of procyclic cells remained firmly connected to the cell body under the same conditions (Figure 2D and 2E). The flagellum did not become detached when cells were first tethered to poly-L-lysine-coated slides (Figure 2F).

**Figure 3:**
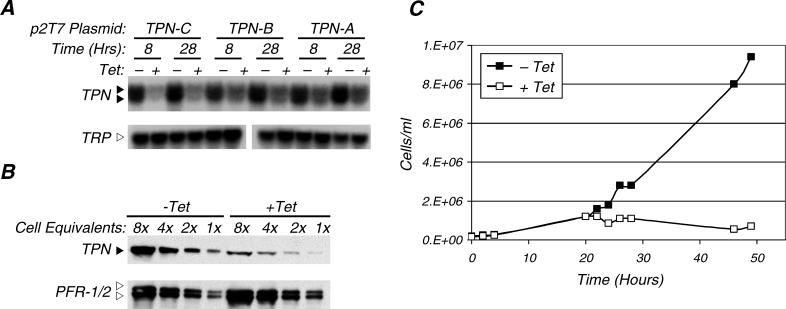
TPN Is Essential in Bloodstream Form Cells (A) BSF-SM cells stably transfected with the indicated p2T7 plasmid (TPN-C, TPN-B, or TPN-A, Materials and Methods) were grown in the presence (+) or absence (−) of tetracycline (Tet). Total RNA samples were prepared 8 h and 28 h after the addition of tetracycline and subjected to northern blot analysis using a TPN probe, or a TRP probe as a loading control (lower blot). The filled arrowheads indicate the position of the TPN mRNA doublet and the open arrowhead indicates the position of TRP mRNA included as a loading control and to demonstrate RNAi specificity. (B) BSF-TPN-C cells were grown in the presence (+) or absence (−) of tetracycline (Tet) for 40 h. Total protein extracts were prepared, serially diluted (8×–1×) and subjected to Western blot analysis using an α-TPN monoclonal antibody. The same blot was re-probed with an α-PFR-1/2 monoclonal antibody to control for protein loading (lower blot). The filled arrowhead indicates the position of TPN and the open arrowheads indicate the positions of PFR-2 (upper band) and PFR-1 (lower band). (C) Growth curve of BSF-TPN-B cells grown in the presence (+ Tet) or absence (− Tet) of tetracycline.

To generate bloodstream form trypanin knockdowns, bloodstream form parasites were stably transfected with a Tet-inducible RNAi plasmid containing one of three different trypanin DNA fragments, trypanin (TPN)-A, B, or C, and clonal cell lines were obtained (Materials and Methods). For clarity, these cell lines are referred to as bloodstream form (BSF)-TPN-A, B, or C, respectively. Northern blot analysis demonstrated that trypanin mRNA was knocked down within 8 h of tetracycline-induced expression of the dsRNA (Figure 3A). Knockdown was most effective using the largest trypanin fragment, TPN-C. We observed a doublet corresponding to trypanin mRNA (Figure 3A, closed arrowheads) and both bands were equally knocked down. Western blots demonstrated that steady-state trypanin protein levels were reduced approximately 4–8 fold following RNAi with TPN-C (Figure 3B).

In procyclic parasites, trypanin knockdown results in loss of directional cell motility, but these mutants remain viable even though trypanin protein is undetectable by Western blot analysis [10]. Surprisingly, all three bloodstream form trypanin knockdown mutants exhibited a severe growth defect. In BSF-TPN-B, this was evident approximately 24 h after the addition of tetracycline (Figure 3C), and by five days post-induction these mutants were not viable. Progression to a lethal phenotype was more rapid in BSF-TPN-C cells and was slowest in BSF-TPN-A cells (unpublished data). Thus, trypanin is essential in the bloodstream lifecycle stage and kinetics of progression to the lethal phenotype correlate with the degree of knockdown.

**Figure 4:**
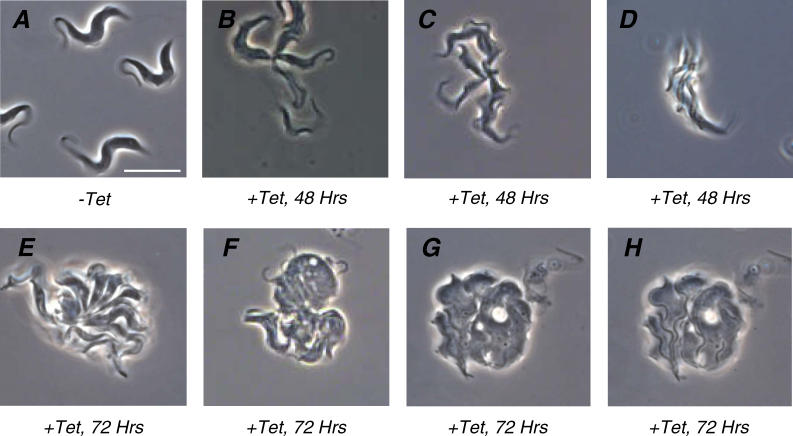
BSF-TPN Mutants Are Defective in Cell Separation (A–H) Phase-contrast images of BSF-TPN-B cells that were grown in the absence (−Tet) (A) or presence (+Tet) (B–H) of tetracycline for the indicated times. (B) and (C) are examples of cell clusters, where four cells are joined together at their extreme posterior tips. (D) is an example of three parallel cells that have failed to undergo cytokinesis. (E) is an example of a very large cell cluster, while (F–H) are examples of amorphous cells that ultimately predominate. The images shown in (G and H) are different focal planes of the same monstrous cell, which is a continuous mass on one face (G) and is clearly multi-flagellated on the other face (H). Scalebar is 10 μm.

**Figure 5:**
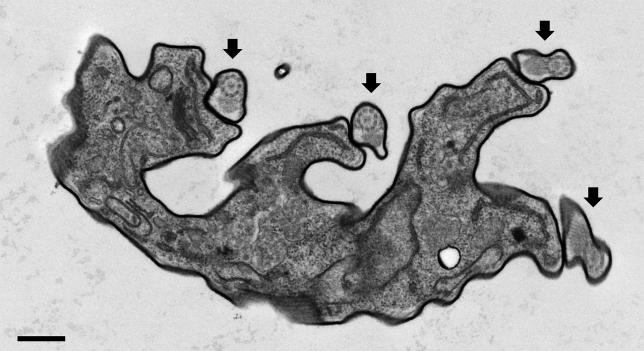
BSF-TPN Mutants Fail in Cytokinesis Transmission electron micrograph of a bloodstream form trypanin knockdown mutant showing a single cell body with multiple (four) flagella (arrows). Scalebar is 0.5 μm.

Close analysis of bloodstream form trypanin knockdown mutants revealed a failure in cytokinesis (Figures 4 and 5). Within 48 h of Tet-induction, clusters of three or more physically attached cells began to appear. Early during the induction, most clusters consisted of three to four cells joined together exclusively at their extreme posterior ends (Figure 4B and 4C). Cytokinesis in T. brucei initiates between the tips of the old and new flagella at the anterior end of the cell [23]. The cleavage furrow then advances along a line between the two flagella until daughter cells are connected just at their posterior ends, with their flagella pointing in opposite directions. This process is best characterized in procyclic cells [23] and the general events are considered to be similar in bloodstream form cells, with the exception that positioning of the kinetoplast, basal body, and nuclei relative to one another differ between lifecycle stages [24,25]. Thus, it seemed that two cells had nearly completed cytokinesis, but remained attached at their posterior, underwent another round of division and again failed to separate. At later time points, clusters were progressively larger and the extent of contact was greater (Figure 4D and 4E). When viewed live under phase-contrast microscopy, individual cell bodies were readily identifiable, but were completely attached to one another along their entire length and appeared to be surrounded by a contiguous membrane. The subpellicular cytoskeleton in T. brucei dictates cell shape and consists of a corset of parallel microtubules that subtend the plasma membrane. This entire structure is normally replicated during each round of cell division. Thus, it appeared as though these cells had replicated their subpellicular cytoskeletons, but had failed to initiate cytokinesis. Ultimately, cells in the culture progressed into large, multi-flagellated masses that were amorphous on one face and multi-flagellated on the other face. Transmission electron microscopy demonstrates that the cytoplasm and cell membrane are contiguous in cells with multiple flagella (Figure 5), demonstrating that they have failed in cytokinesis.

### Trypanin Mutants Proceed through Multiple Rounds of Organelle Replication

**Figure 6:**
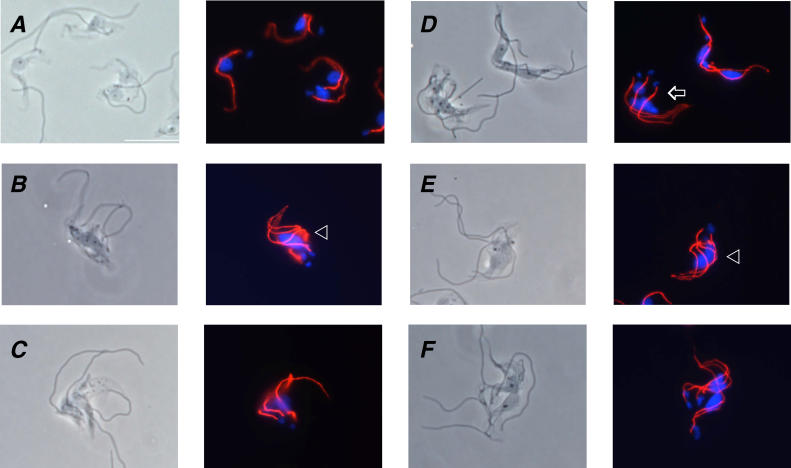
FAZ Replication Is Not Sufficient to Drive Cytokinesis in Bloodstream Form Parasites Immunofluorescence analysis of BSF-TPN-B cells grown in the absence (A) or presence (B–F) of tetracycline for 24 h. Cytoskeletons were prepared by detergent extraction and used for immunofluorescence with α-FAZ monoclonal antibodies. Phase-contrast images are shown in the left panels and merged images are shown in the right panels, with antibody staining in red and DAPI staining in blue. Arrowheads indicate examples of cells with more than two flagella and a single diffuse nucleus. Arrows indicate examples of cells with more than two flagella and two or more nuclei. Scalebar is 10 μm.

Recent work has demonstrated that trypanosomes possess novel cell cycle checkpoints and suggests that replication and segregation of the flagellum, FAZ, kDNA, and nuclear DNA are pivotal events in cell cycle progression [14,15,26–28]. We therefore examined these cellular structures in bloodstream form trypanin knockdown mutants (Figure 6). The order of events for organelle replication during the cell division cycle in T. brucei has been described in detail [11,23,24,29] and is briefly summarized here. At the start of the cell cycle, G1 cells contain a single kinetoplast, flagellum, FAZ, and nucleus. The first event observed is maturation of the pro-basal body, which nucleates a new flagellum that emerges from the flagellar pocket posterior to the existing flagellum. The kinetoplast is subsequently replicated and divides, with the new kinetoplast moving posterior to the old kinetoplast while the new flagellum extends toward the anterior end of the cell, parallel to the existing flagellum. A FAZ subtends each flagellum along its length. Following kinetoplast division and flagellum elongation, mitosis ensues to yield a single cell with two copies each of the kinetoplast, flagellum, FAZ, and nucleus. At this point, cytokinesis initiates between the tips of the flagella at the anterior end of the cell. The cleavage furrow progresses unidirectionally from anterior to posterior to yield two daughter cells, each having a single kinetoplast, flagellum, FAZ, and nucleus.

**Figure 7:**
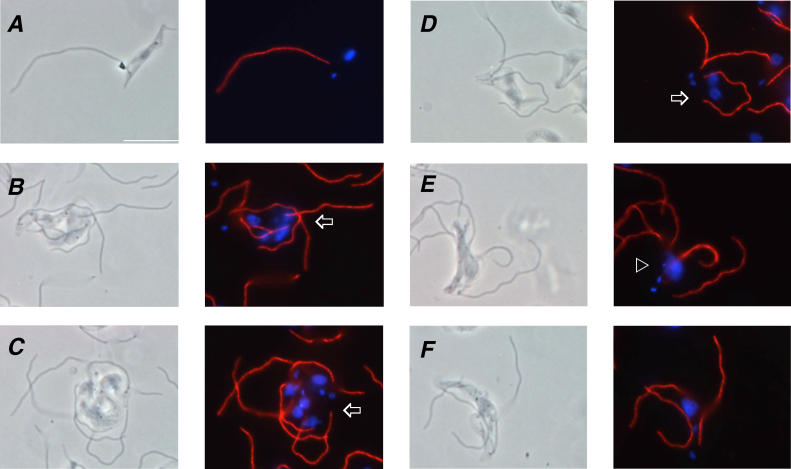
BSF-TPN Mutants Proceed through Multiple Rounds of Flagellum Replication, but Ultimately Fail to Initiate Cytokinesis Immunofluorescence analysis of BSF-TPN-B cells grown in the absence (A) or presence (B–F) of tetracycline for 24 h. Cytoskeletons were prepared by detergent extraction and used for immunofluorescence with α-PFR-2 (A–F) monoclonal antibodies. Phase-contrast images are shown in the left panels and merged images are shown in the right panels, with antibody staining in red and DAPI staining in blue. Arrowheads indicate examples of cells with more than two flagella and a single diffuse nucleus. Arrows indicate examples of cells with more than two flagella and two or more nuclei. Scalebar is 10 μm.

**Figure 8:**
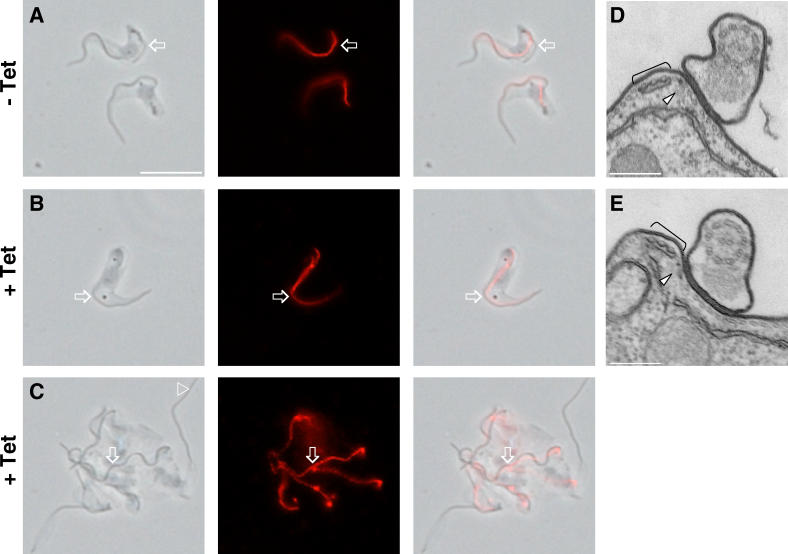
The Flagellum Is Properly Positioned Next to the FAZ in BSF- TPN Mutants Cells were cultured in the absence (− Tet, A and D) or presence (+ Tet, B, C, and E) of tetracycline, then processed for immunofluorescence (A–C) or transmission electron microscopy (D–E). (A–C) Live cells were first adhered to poly-L-lysine-coated coverslips to reduce flagellum detachment and then processed for immunofluorescence using mAb L3B2 to visualize the FAZ (red). Flagella can be seen clearly in phase contrast images (arrows) and, where still attached to the cell body, the flagellum runs parallel to the FAZ (red). In some cases, the flagellum becomes detached from the cell (open arrowhead, panel C), as noted earlier (see text). (D–E) Transmission electron microscopy shows that the flagellum is properly positioned adjacent to the FAZ, which is marked by a gap between the subpellicular microtubules (arrowhead) and a set of reticulum-associated microtubules (bracket) immediately adjacent to this gap [6]. Note that the image in E was taken from a cell that had failed cytokinesis and had at least three flagella. Scalebars are 10 μm (A–C), 0.25 μm (D–E).

Immunofluorescence with antibodies against the FAZ revealed that trypanin knockdown mutants contained up to six distinguishable, parallel FAZ structures (Figure 6). Wild-type cells (Figure 6A) possess only one FAZ or, during cell division, two that extend in parallel from adjacent to the kinetoplast toward the anterior tip of the cell. The multiple FAZ structures in the mutants appear to be placed correctly and extend in parallel to the anterior end of the cell (Figure 6B–6F). Thus, formation and extension of the FAZ, although critical for positioning of the site of cell cleavage in procyclic culture form (PCF) parasites [14], is not sufficient to allow cytokinesis in bloodstream form cells. Similarly, flagellum replication is unaffected by loss of trypanin (Figure 7). Knockdown mutants frequently contained six distinguishable flagella (Figure 7C), and in some cases as many as 13 were visible (unpublished data). Wild-type cells contain only one flagellum (Figures 2B and 7A) or two flagella during cell division [23]. To determine whether placement of the flagellum is normal in trypanin knockdown mutants, cells were first adhered to poly-L-lysine-coated slides (Figure 2F), allowing us to maintain flagellum attachment during immunofluorescence. As shown in Figure 8, the flagellum extended parallel to the FAZ along the cell body in knockdown and uninduced cells. The flagellum was also properly positioned adjacent to the FAZ when whole cells were examined by transmission electron microscopy (Figure 8D, E) and was placed normally in phase-contrast images of live cells (Figure 4). Therefore, the flagella of trypanin knockdown mutants were positioned correctly along the FAZ.

**Figure 9:**
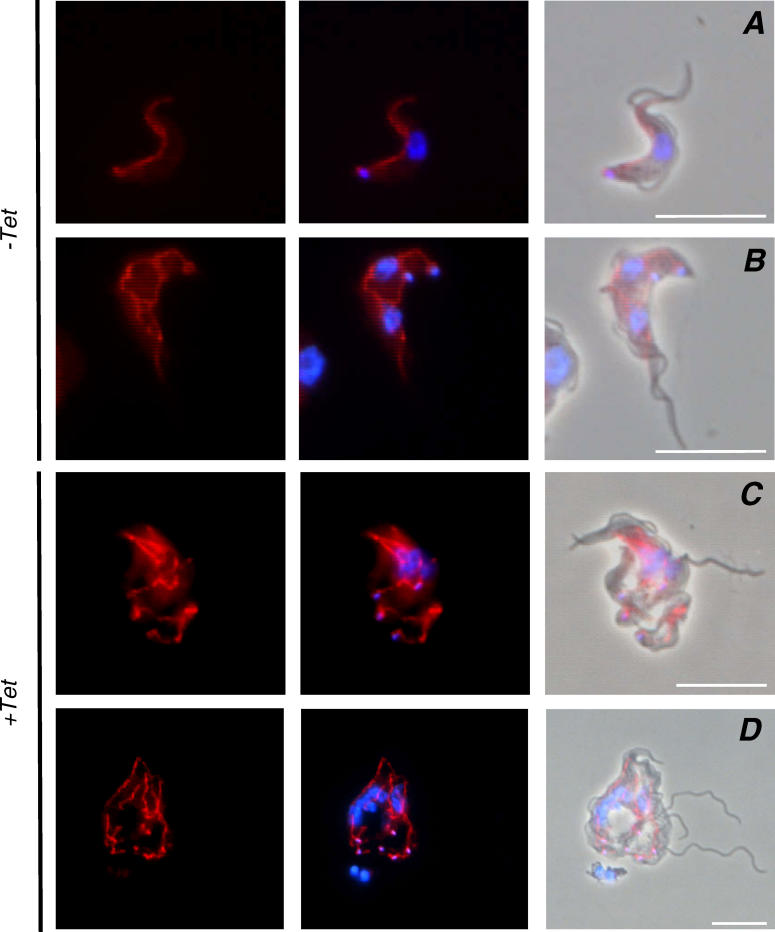
Mitochondrial Replication Continues in BSF-TPN Knockdown Mutants Cells were cultured in the absence (− Tet) or presence (+ Tet) of tetracycline and mitochondria were visualized with mitotracker. (A–B) The mitochondrion of uninduced cells replicates as described for wild type trypanosomes [29]. (A) shows a G1 cell. The mitochondrion is a single ribbon on the side of the cell opposite the flagellum with a focal point of staining at the kinetoplast. (B) shows a post-mitotic cell. The nucleus, flagellum and kinetoplast have replicated. The mitochondrion is branched with focal points of staining at the two kinetoplasts. (C and D) show representative Tet-induced trypanin knockdowns. There are multiple mitochondrial ribbons, each with a focal point of staining at the kinetoplasts.

We used 4′,6 diamidino-2-phenylindole (DAPI) staining to monitor replication of the nucleus and kinetoplast (Figures 6 and 7). Although wild-type cells contain one nucleus and one kinetoplast, or up to two nuclei and two kinetoplasts during cell division [30], trypanin mutants frequently contained multiple nuclei and kinetoplasts or a single diffuse nucleus with several kinetoplasts (Figures 6 and 7). In almost all cases, kinetoplasts were well-resolved from one another and initiated assembly of a new flagellum, indicating that kinetoplast segregation and basal body function were normal. Blocking mitosis in bloodstream form trypanosomes prevents cytokinesis [26,27]. We were therefore particularly interested in determining whether the block in cytokinesis in trypanin knockdown mutants correlated with mitotic failure. In some cases, mitosis appeared to have failed, as indicated by the presence of more than two flagella and a single diffuse nucleus (arrowheads, Figure 6B and 6E). However, there were also several examples of cells having more than two flagella and two or more nuclei (arrows, Figure 6D, 7B–7D), indicating that they had undergone at least one round of mitosis without cytokinesis and then continued through another round of flagellum replication. This result supports the finding that mitosis can occur in the absence of cytokinesis in bloodstream form trypanosomes [17,31]. Mitochondrial replication [29] also continued unchecked in trypanin knockdown mutants (Figure 9).

## Discussion

We have demonstrated that trypanin, a component of an evolutionarily-conserved dynein regulatory system [12,22], is localized to the flagellum of bloodstream form trypanosomes and is essential in this lifecycle stage. An essential role for trypanin in the bloodstream stage is surprising, since procyclic-form, trypanin knockdown mutants are viable [10]. Trypanin is quantitatively associated with the flagellar apparatus in procyclic [10,21] and bloodstream form (Figure 2) cells and its only known role is in the coordinate regulation of axonemal dynein [10,12,22]. Phylogenetic analysis indicates that trypanin is found only in organisms that assemble motile flagella, consistent with its role in regulation of flagellar beat. Therefore, our results suggest that regulation of flagellar beat is essential in bloodstream form African trypanosomes.

We cannot rule out the formal possibility that trypanin has another, as yet unknown, role in the cell and that loss of viability is due to something other than trypanin's role in beat regulation. However, biochemical and immunofluorescence studies demonstrate it is exclusively associated with the flagellar apparatus in bloodstream (Figure 2) and procyclic [10,21] *T. brucei,* making a role outside the flagellum unlikely in these parasites. It is possible that the cell senses fidelity of the flagellar apparatus and that reduction in trypanin protein levels, rather than a motility defect per se, is lethal. However, since loss of trypanin does not cause gross changes in axoneme ultrastructure in bloodstream (Figures 5 and 8) or procyclic [10] cells, we consider this possibility less likely.

To our knowledge, this is the first time that a defined dynein regulatory complex has been demonstrated to be essential in any organism. These findings support our previous demonstration of a role for flagellar motility in cytokinesis in procyclic trypanosomes [12] and implicate the DRC and other enzymatic regulators of flagellar motility as candidate drug targets for the treatment of African sleeping sickness. We further show that the cell division defect in bloodstream form cells is not due to abnormal replication of the FAZ, which serves as an important structural guide for cleavage furrow formation in procyclic cells [14,32]. Enzymes and ligand-binding regulatory proteins comprise a large fraction of proteins considered to be good drug targets [33,34]. Hence an essential role for flagellar beat, which requires numerous enzymatic and regulatory activities [35], would greatly extend the potential of the flagellum as a drug target. In agreement with this idea, during preparation of this manuscript, Gull and co-workers also reported an essential role for flagellar proteins identified in a T. brucei extracted axonemal proteome [36]. A critical next step in exploiting these findings for potential development of novel chemotherapeutic strategies will be isolation of key enzymes necessary for flagellar motility, accompanied by structural studies to facilitate drug discovery. As part of a defined signaling complex that regulates axonemal dynein [10,12,22,35], the DRC represents an excellent starting point for these studies.

At present, the precise mechanism by which trypanin, and by extension flagellar beat, contributes to cytokinesis is not clear. In procyclic cells, it appears that cytokinesis failure reflects, at least in part, a direct requirement for flagellar beat during cell cleavage [12]. However, as noted below, the end-stage phenotypes of procyclic mutants and bloodstream mutants differ significantly. Abnormal flagellar beat might also disrupt replication or positioning of the flagellum, FAZ or kinetoplast [12,37], all of which are important for cytokinesis [14,25,32]. We find that these organelles are clearly replicated in bloodstream form trypanin knockdown mutants and appear to occupy their normal cellular positions. Likewise, these mutants often contain multiple nuclei, indicating that they can complete multiple rounds of mitosis. Therefore, the cytokinesis defect is not simply the indirect consequence of failed organelle replication or positioning.

We previously demonstrated that active flagellar beating is required for cell division in procyclic-form trypanosomes [12] and our current results extend this finding to bloodstream form parasites. However, there are two important differences between these lifecycle stages. First, the requirement for flagellar beat appears to be more stringent in bloodstream form trypanosomes, since trypanin knockdown is lethal in the bloodstream form, whereas procyclic trypanin knockdown mutants are viable and capable of cell separation [10]. The simplest interpretation of this result is that although severe flagellar beat defects impair division in procyclic cells, abnormal beat regulation is tolerated, whereas even modest perturbation of flagellar beat is lethal in bloodstream form parasites. A second notable difference is that procyclic motility mutants fail during the progression and/or completion of cell cleavage, accumulating as clusters of cells attached at their posterior tips [12]. In contrast, bloodstream form trypanin mutants ultimately fail at a much earlier stage of cell division. Analysis of kinetics of phenotype progression in bloodstream form trypanin mutants revealed that failure to initiate cytokinesis is preceded by a failure at the late-stages of cell separation. However, these mutants ultimately fail to initiate cytokinesis and accumulate as amorphous masses with multiple nuclei, kinetoplasts and flagella. These results emphasize the dichotomy between lifecycle stages with respect to factors that contribute to cell division and cell morphogenesis [26,27].

Previous studies have demonstrated that there are differences in cell cycle regulation in procyclic versus bloodstream form trypanosomes [26,27], and our work demonstrates that these differences extend to physical aspects of cell division. Whether there is a connection between flagellar beat and signaling events that control cell cycle progression remains to be determined, though the recent report of a polo-like kinase localized at or near the flagellum attachment zone [38] make this a tantalizing speculation. The T. brucei flagellum is connected along its length to the cell body, such that flagellar movements are transmitted directly to the plasma membrane. Hence, flagellar beating and the concomitant forces exerted on the plasma membrane and cell body might be necessary to facilitate membrane and cytoskeletal rearrangements that accompany replication and separation of subpellicular microtubules. Clearly, many more exciting discoveries can be expected from continued efforts to ascertain the exact nature of the connection between flagellar beat and cell division in these deadly pathogens.

## Materials and Methods

### Cell culture and transfection.

BSF-SM cells [39] and procyclic 29–13 cells [39], which stably express T7 polymerase and Tet repressor, were used for all experiments. Procyclic cells were cultured as described [21]. BSF-SM cells were cultivated at 37 °C and 5% CO_2_ in HMI-9 medium supplemented with 10% heat-inactivated fetal calf serum. Transfections were performed using an adaptation of the method described by [17]. Briefly, cells were transfected with *Eco* RV-linearized plasmids and stably-transfected clonal cell lines were recovered by selection with 5 μg/ml phleomycin in a 24-well plate. For tetracycline induction, cells were split into two flasks and cultured with or without tetracycline (300 ng/ml). For visualizing mitochondria, cells were labeled 24-h post-induction with 10 nM mitotracker CMX Ros (Molecular Probes, Eugene, Oregon, United States) for 30 min, then harvested, washed twice, and re-suspended in fresh medium. Following a 40-min chase period, cells were harvested, washed twice in cold PBS, spotted on to poly-L-lysine-coated coverslips, fixed with paraformaldehyde, stained with DAPI, and visualized by fluorescence microscopy.

### DNA constructs.

To create TPN RNAi plasmids, three different fragments of TPN were cloned into p2T7-Ti-B/GFP [17], replacing the GFP fragment between the opposing tetracycline-inducible T7 promoters. The p2T7-TPN-A and p2T7-TPN-B plasmids contain adjacent TPN fragments of similar sizes. p2T7-TPN-A contains a 273-nt fragment of TPN that corresponds to nt 829-1102 of the TPN ORF. p2T7-TPN-B contains a 251-nt fragment of TPN that corresponds to nt 1103–1354 of the TPN ORF. The p2T7-TPN-C plasmid contains a 525-bp fragment (nt 829-1354 of the TPN ORF) that precisely spans both the TPN-A and the TPN-B fragments.

p2T7-TPN-A and p2T7-TPN-B were cloned by amplification of nts 829-1354 of the TPN ORF with the following primers: 5′-CGGGATCCTGTTGCACCGTTAGAAGAAGC-3′ and 5′-CGGGATCCGTTGCTACGTGGCAGTGAAG-3′ (underlined bases denote *Bam* HI sites) with Pfu polymerase (Invitrogen, Carlsbad, California, United States). This PCR product was cloned into pCR-Blunt-II-TOPO (Invitrogen), and the TPN-A and TPN-B fragments were subcloned into *Bam* HI/*Hind* III in p2T7-Ti-B/GFP using the introduced *Bam* HI sites and an internal *Hind* III site at nt 1102 in the TPN ORF.

p2T7-TPN-C was cloned by amplification of nts 829-1354 of the TPN ORF with the following primers: 5′-TGCTCTAGATGTTGCACCGTTAGAAGAAGC-3′ (underlined bases denote an *Xba* I site) and 5′-TCCCCGCGGGTTGCTACGTGGCAGTGAAG-3′ (underlined bases denote a *Sac* II site) with Pfu polymerase. This PCR product was cloned into pCR-Blunt-II-TOPO and the TPN-C fragment was subcloned into *Xba* I/*Sac* II in p2T7-Ti-B/GFP using the introduced *Xba* I and *Sac* II sites.

### Protein sequence alignments and phylogenetic analysis.

Protein sequence alignments were performed using the Align X program in Vector NTI Advance (Informax, Frederick, Maryland, United States), which utilizes the Clustal W algorithm [40]. Phylogenetic tree assembly is based on the sequence distance method and utilizes the Neighbor Joining (NJ) algorithm [41]. Note that kinetoplastids contain a second trypanin-related protein [12], which was excluded from this phylogenetic analysis for simplicity.

### RNA preparation and northern blotting.

Total RNA samples were prepared using an RNeasy kit (Qiagen, Valencia, California, United States) according to the manufacturer's instructions. RNA samples (5 μg) were analyzed by northern blotting as described previously [42]. ^32^P-labeled probes corresponding to nt 1–775 of the TPN ORF or the entire TRP ORF were used for northern blotting analysis. The TRP gene (GeneDB ID Tb09.244.2800) encodes a trypanin-related protein with 28% identity and 43% similarity to trypanin and will be described elsewhere (K. L. Hill, unpublished data).

### Protein preparation and western blotting.

Protein extracts were prepared and analyzed by western blotting [21,43]. For serial dilutions, protein extracts were serially diluted in two-fold increments, and 1.25 × 10^6^ cell equivalents per lane to 1.56 × 10^5^ cell equivalents per lane were used for western blotting analysis. Trypanin was detected with a monoclonal antibody directed against a synthetic peptide corresponding to the last 13 amino acids of trypanin and was generated by an outside vendor (Cell Essentials, Cambridge, Massachusetts, United States). Blots were then re-probed with α-PFR-1/2 L13D6 [44] as a control for protein loading. Fold-differences in TPN abundance between uninduced and Tet-induced cells were estimated by comparing the band intensity of the serially diluted samples.

### Immunofluorescence and electron microscopy.

Cells were split into two flasks and cultured with or without tetracycline for 24 h. Cytoskeletons were prepared for immunofluorescence by detergent-extraction as described previously [21,45]. Primary antibodies were added at a 1:50 dilution (α-TPN, α-PFR-2 L8C4, or α-FAZ L3B2 monoclonal antibodies) [44]; α-mouse secondary antibodies conjugated to Alexa-Flor488 (Molecular Probes) were added at a 1:400 dilution. Samples were mounted in vectashield (Vector Labs, Burlingame, California, United States) and imaged on a Zeiss Axioskop II compound microscope. Transmission electron microscopy was done as described previously [10] on samples prepared 28 h post-induction.

## Supporting Information

### Accession Numbers

The NCBI GenBank (http://www.ncbi.nlm.nih.gov) and GeneDB (http://www.genedb.org) accession numbers included in this study are *D. melanogaster* (NC 004354), A. gambiae (EAA11363), H. sapiens (NM 001481), M. musculus (AY692443), C. reinhardtii (AAP57169), L. major (LmjF36.1910), T. cruzi (Tc00.1047053507641.210), T. brucei (O15697), G. lamblia (EAA37004). S. mansoni (AAX28498), D. ranerio (AAH57501), C. intestinalis (ci0100145616), S. purpuratus (XP_782050), and G. gallus (BX932390).

## Abbreviations

BSF: bloodstream form
DAPI: 4′, 6 diamidino-2-phenylindole
DRC: dynein regulatory complex
FAZ: flagellum attachment zone
PCF: procyclic culture form
PFR: filamentous paraflagellar rod
RNAi: RNA interference
Tet: tetracycline
TPN: trypanin
TRP: trypanin-related protein
